# Comparing the accuracy of ultrasound-based measurements of the cervical vagus nerve

**DOI:** 10.1038/s41598-023-27894-9

**Published:** 2023-01-17

**Authors:** Johann Dörschner, Johann Otto Pelz, Alexander Michael Kerner, Jason John Labuschagne, Niels Hammer, Sabine Löffler

**Affiliations:** 1 Department of Anatomy, University of Leipzig, Leipzig, Germany; 2 Department of Neurology, University of Leipzig, Leipzig, Germany; 3 Division of Macroscopic and Clinical Anatomy, Gottfried Schatz Research Center, Medical University of Graz, Graz, Austria; 4 Department of Neuroscience, University of the Witwatersrand, Johannesburg, South Africa; 5 Department of Orthopedic and Trauma Surgery, University of Leipzig, Leipzig, Germany; 6 Division of Biomechatronics, Fraunhofer Institute for Machine Tools and Forming Technology, Dresden, Germany

**Keywords:** Autonomic nervous system, Epilepsy

## Abstract

Vagus nerve stimulation (VNS) has become a promising therapy especially for drug resistant epilepsy and other pathologies. Side effects or missing therapeutic success are observed due to cuff electrodes that are too narrow or too wide. Preoperative high-resolution ultrasound is used to evaluate the size of the cervical vagus nerve (CVN) to estimate the size of cuff electrodes for VNS. It remains unclear how precise ultrasound reflects the CVN dimensions, which has been the objective of this study. CVN cross-sections and diameters were investigated in 23 sides from 12 bodies, using ultrasound, histology, and CVN casting in situ as a reference. Morphometric data were obtained including fascicle count and nerve composition in histology. CVN yielded significant side-, age-, and BMI-related differences. CVN cross-sections were smaller in ultrasound when compared to casting and histology (1.5 ± 0.4 vs. 3.1 ± 0.9 vs. 2.3 ± 0.7 mm^2^). With the given setting in ultrasound, CVN cross-sections were consistently underestimated when compared to casting. Ultrasound-based cross-section measurements are related to a biased estimation of CVN size. A factor to correct for method related differences may help to adjust for accurate cuff electrode sizes for patient needs and to reduce undesired effects and potentially material consumption.

## Introduction

The cervical part of the vagus nerve (CVN) has become a structure of interest for vagus nerve stimulation (VNS) which has evolved to be a promising therapy for drug resistant epilepsy^1–7^ and other pathologies such as rheumatoid arthritis^8,9^. When implanting a cuff electrode for VNS, the CVN is first localized within the carotid sheath^10^. A helical cuff electrode is directly wrapped around the CVN^11^ and connected to an impulse generator placed in a sub-clavicular pocket^12–14^. While the surgical procedure is described to be manageable and safe^15^, two of three patients experience side effects^16^ and 25.4% of all patients receive no measurable benefit at all from VNS^3^. Previous studies focused on morphological investigations describing the chosen body side^7,17^, the variable position of the nerve within the carotid sheath^10^, nerve branching^18^, and complex vascularity^11^ as potential impacts on the surgical approach for VNS. Known as a non-invasive procedure, (high-resolution) ultrasound is an established diagnostic tool for neurological pathologies such as nerval degeneration or neuropathy^19–22^. Ultrasound could be used preoperatively for morphological evaluation and cross-sectional measurements of the CVN, which may help to estimate the matching size of cuff electrodes for VNS. However, it is unclear until now how precise ultrasound reflects the CVN dimensions. Multiple ultrasound laboratories have reported reference values for the CVN with mean cross-sectional areas (CSAs) ranging widely between 2.2 and 5.7 mm^2^^19,23–25^. Few authors have reviewed ultrasound measurements with manual measurements of nerval tissue^26,27^. Studies reviewing ultrasound values for the CSA of the CVN with manual measurements have not been published yet. Further knowledge on the accuracy of the CVN in ultrasound compared to manual measurements is needed to potentially improve the surgical approach and the implantation of cuff electrodes. The aim of this study was to critically revisit preoperative ultrasound as guidance for VNS surgery to improve the safety and efficiency of implanting cuff electrodes. Therefore, the CVN CSA of unembalmed *post-mortem* bodies was measured in ultrasound and histology and compared to direct casting as a reference, which had been performed in a similar manner to the surgical exposure as done with patients that undergo electrode implantation for VNS. Morphologically relevant data such as fascicle count, substructural composition, and vascularity of the CVN were recorded as an anatomical basis for VNS. Surprisingly, the CSA ranged widely depending on the method of measurement. The following questions have been addressed:

1. Does preoperative high-resolution ultrasound of the CVN CSA accurately reflects the nerve’s true size?
2. How can method-based differences on the measured CSA be explained and can the error be adjusted for?

## Results

### The CVN yields side, age, and BMI related CSA differences

The average CSA on the right side was larger when compared to the left side. This difference was present in the casts with epineurium (*p* = 0.012), and in histology with (*p* = 0.010) and without (*p* = 0.017) epineurium. Therefore, further measurements were made in consideration of the body side. Age correlated negatively with the CSA derived from casting with epineurium on the right side (r = − 0.535, *p* = 0.037). Correlations were present for body mass index (BMI) with ultrasound with epineurium (r = 0.609, *p* = 0.011), and for BMI with casting with epineurium (r = 0.54, *p* = 0.035) on the right side. Histological analyses yielded the overall CSA consisting of epineurium, stroma and axon tissue. Strong inverse correlations between the proportions of epineurium and the proportion of axon tissue at overall CSA on both sides (r ≤ − 0.726, *p* ≤ 0.006) were shown. BMI seemed to influence the proportion of epineurium at overall CSA positively (r = 0.653, *p* = 0.011), while BMI negatively influenced the proportion of axon tissue at overall CSA (r = − 0.609, *p* = 0.018) for the right CVN. The supportive tissue in between the nerve fascicles yielded no correlation with BMI (*p* ≥ 0.347).

### Greater and lesser CVN diameters depend on the method

In ultrasound, the mean greater diameter was 1.4 ± 0.3 mm (mean ± standard deviation; left) and 1.8 ± 0.4 mm (right), the lesser diameter averaged 0.9 ± 0.1 mm (left) and 1.0 ± 0.2 mm (right), respectively, both within a hyperechoic rim. Greater diameters in casting with epineurium averaged 2.0 ± 0.3 mm (left) and 2.2 ± 0.5 mm (right), lesser diameter averaged 1.4 ± 0.2 mm (left) and 1.6 ± 0.3 mm (right). In histology, greater CVN diameter including the epineurium averaged 1.8 ± 0.5 mm (left) and 2.1 ± 0.4 mm (right), lesser diameter averaged 1.1 ± 0.3 mm and 1.4 ± 0.2 mm (right), respectively.

### CVN composition, vascularity, and fascicle count

**Figure 1:**
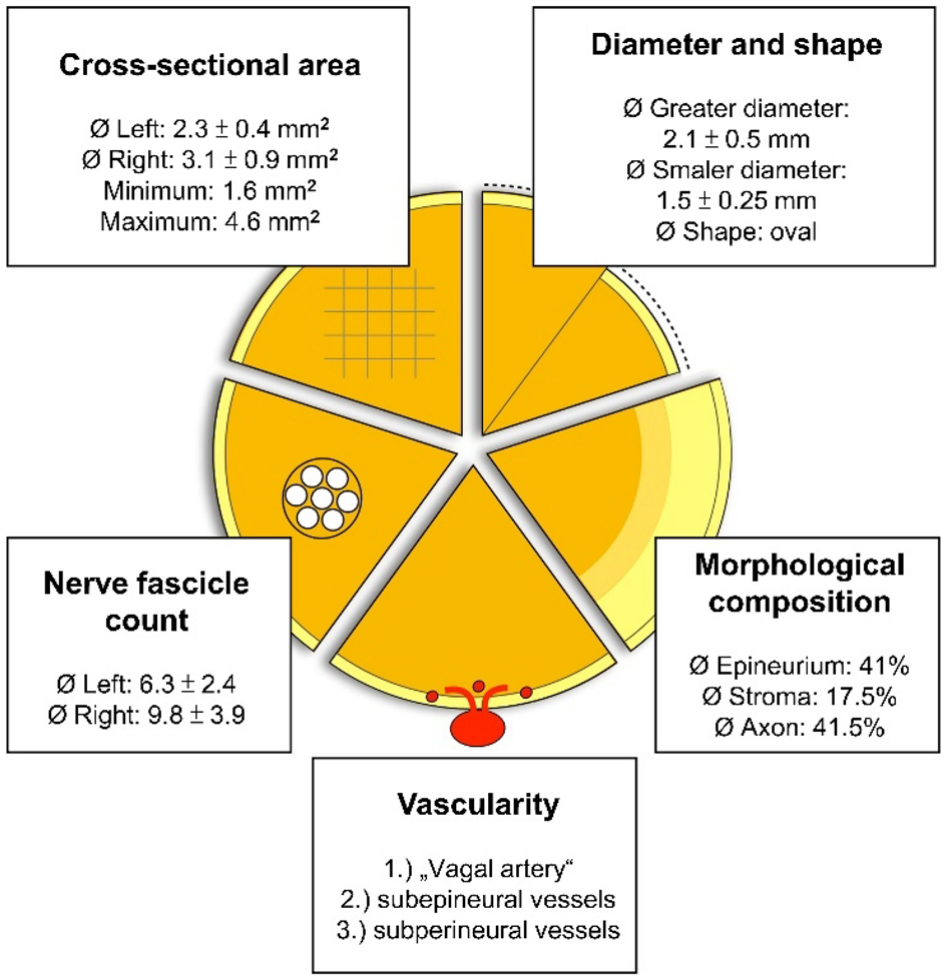
Overview on morphological findings derived from casting (cross-sectional area, diameter, and shape) and histology (nerve fascicle count, vascularity, and morphological composition; results with standard deviation; for diameter, shape, and morphological composition the average of both body sides is given).

**Figure 2:**
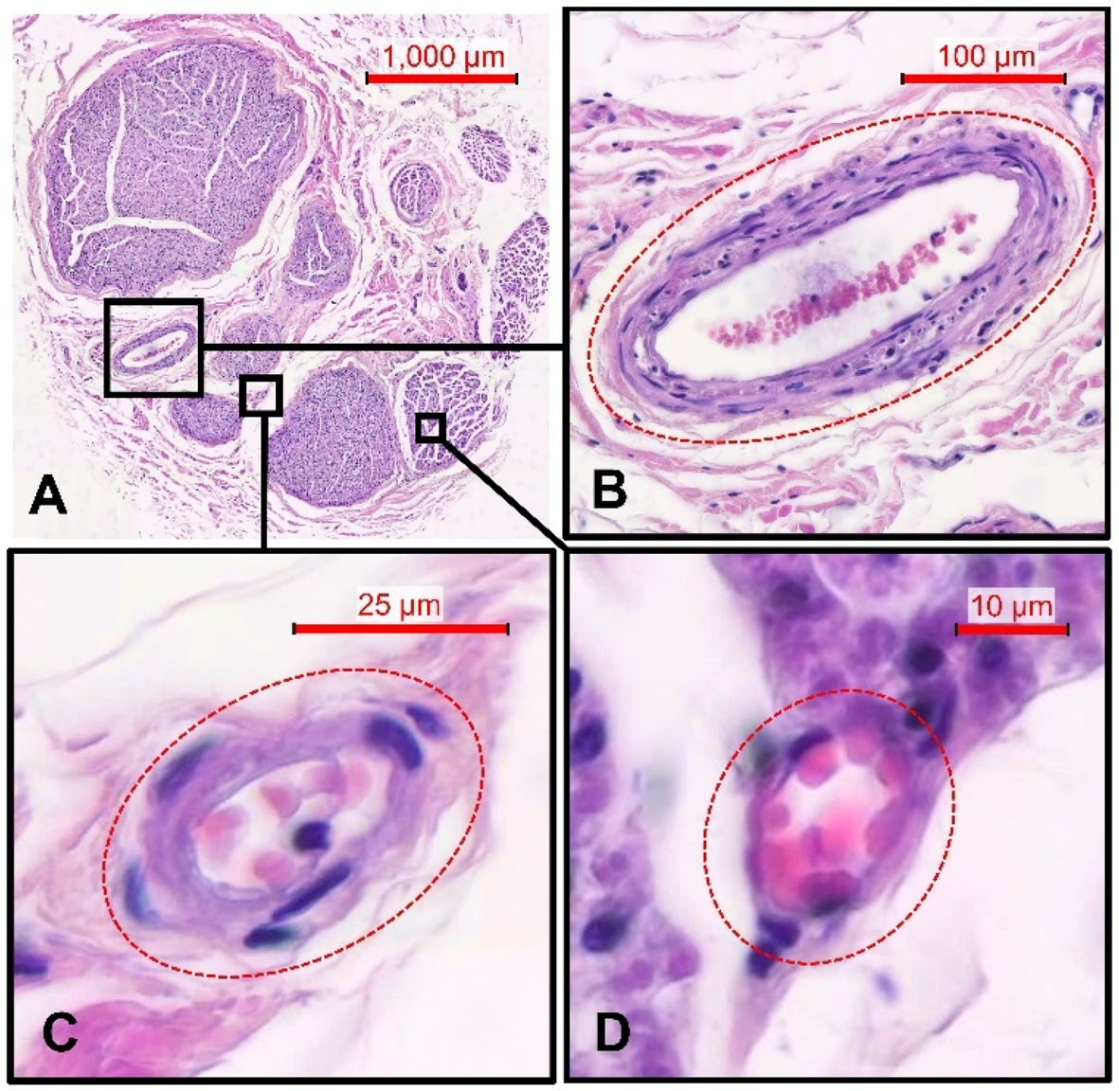
Histology sample of the cervical vagus nerve stained with hematoxylin–eosin. Nerve fascicles surrounded by perineurium and epineurium can be seen (**A**). Vascular supply with the “vagal artery” laying external to the epineurium **(B)**, subperineural vessels within the perineurium (**C**), and minor vessels within the axon tissue within the nerve’s fascicles (**D**) are shown. Pictures of (**B**–**D**) are magnifications of (**A**) in given resolution.

On the average the, CSA of the CVN consists of 41.0% epineurium, 17.5% stroma, and 41.5% axon tissue measured in histology (Fig. 1). Furthermore, the number of fascicles was different between the left and the right body side. While the left CVN consisted of 6.3 fascicles, the right nerve yielded 9.6 fascicles in average. Vascularity of the CVN was illustrated in one case (Fig. 2), showing greater and smaller vessels supplying the nerve. Magnifications (B-D) depict the vessels’ cross-sections in given resolutions containing erythrocytes. Vascularity was observed to be organized in greater vessels lying external to the epineurium, in smaller vessels between the nerve’s fascicles within the perineurium, and in minor vessels within the axon tissue within the nerve’s fascicles.

### The CSA of the CVN largely depends on the method of measurement

Significant difference was determined for the CSA of the CVN using different methods (*p* ≤ 0.02). The CSA measured with ultrasound averaged 1.2 ± 0.4 (range 0.7 to 1.7) mm^2^ for the left and 1.5 ± 0.4 (range 0.8 to 2.3) mm^2^ for the right side, respectively. Casting with epineurium yielded CSA values of 2.3 ± 0.4 (range 1.6 to 3.0) mm^2^ for the left and 3.1 ± 0.9 (range 2.1 to 4.6) mm^2^ for the right side; histology 1.5 ± 0.5 (range 0.7 to 2.7) mm^2^ for the left side and 2.3 ± 0.7 (range 1.6 to 3.8) mm^2^ for the right side, respectively (Table 1).

**Table 1:** Greater and lesser diameters of the cervical vagus nerve depend on the method of measurement.

n = 23	Greater diameter ultrasound	Lesser diameter ultrasound	Greater diameter cast with epineurium	Lesser diameter cast with epineurium	Greater diameter histo with epineurium	Lesser diameter histo with epineurium
	(mm)	(mm)	(mm)	(mm)	(mm)	(mm)
All	1.6 ± 0.4	1.0 ± 0.2	2.1 ± 0.4	1.5 ± 0.3	2.0 ± 0.5	1.3 ± 0.3
Maximum	2.9	1.6	3.5	2.0	3.2	1.8
Minimum	0.9	0.7	1.5	1.0	1.1	0.6
Left	1.4 ± 0.3	0.9 ± 0.1	2.0 ± 0.3	1.4 ± 0.2	1.8 ± 0.5	1.1 ± 0.3
Right	1.8 ± 0.4	1.0 ± 0.2	2.2 ± 0.5	1.6 ± 0.3	2.1 ± 0.4	1.4 ± 0.2

Values given in mm. Results with standard deviation. The sample size is indicated by n.

**Figure 3:**
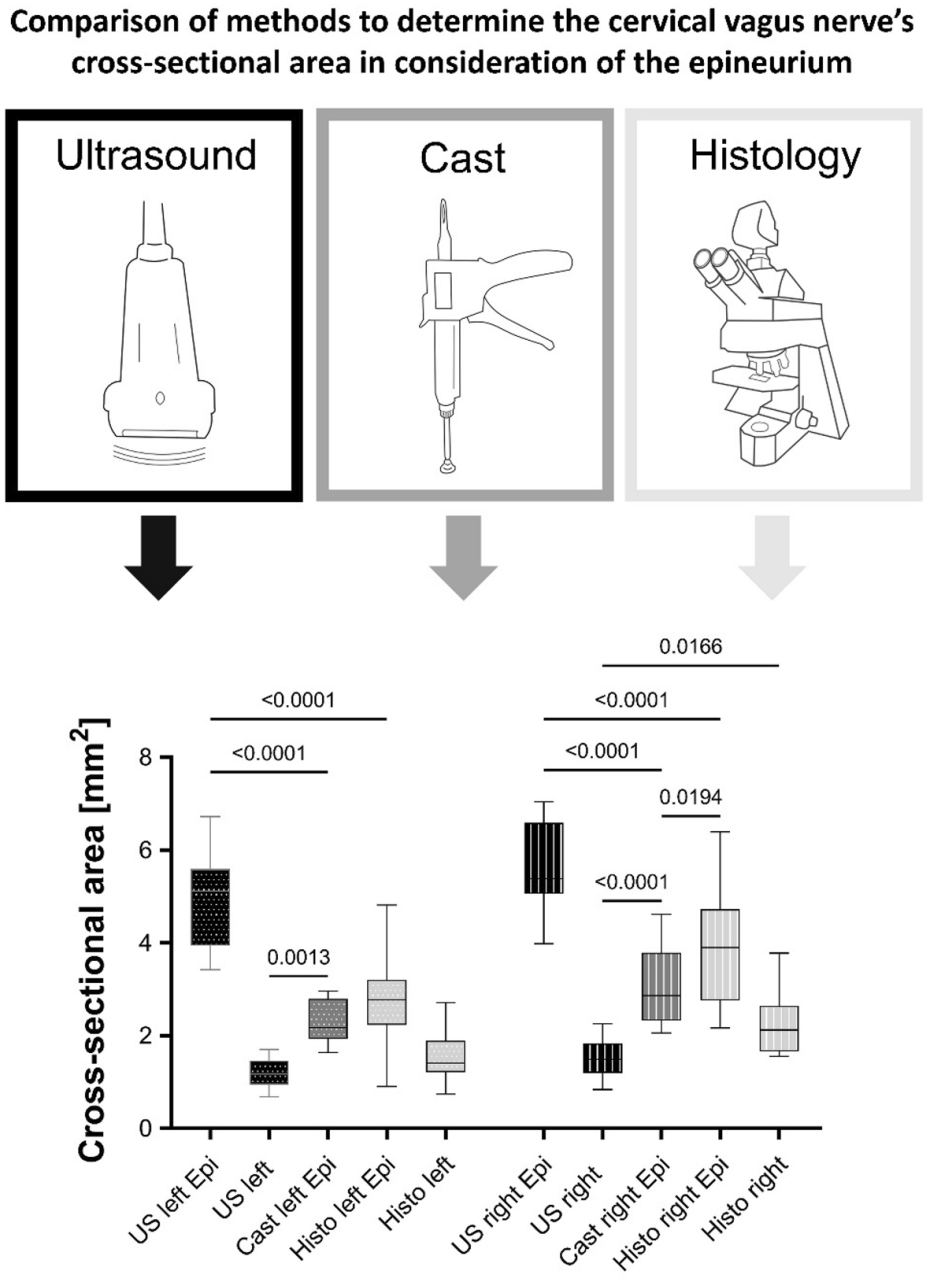
Box plots comparing different methods to determine the cervical vagus nerve’s cross-sectional area in consideration of the epineurium. Dotted Boxes indicate the left side, striped boxes the right side. The boxes show the 25th, 50th and 75th percentile, whiskers the minima and maxima. The solid line marks the median. Black—ultrasound (US), darker grey—cast, lighter grey—histology (Histo); Epi: Measurements contain the epineurium.

Considering the appearance of the epineurium in ultrasound and casting, measurements were adapted, differentiating between with or without epineurium (Fig. 3).

**Figure 4:**
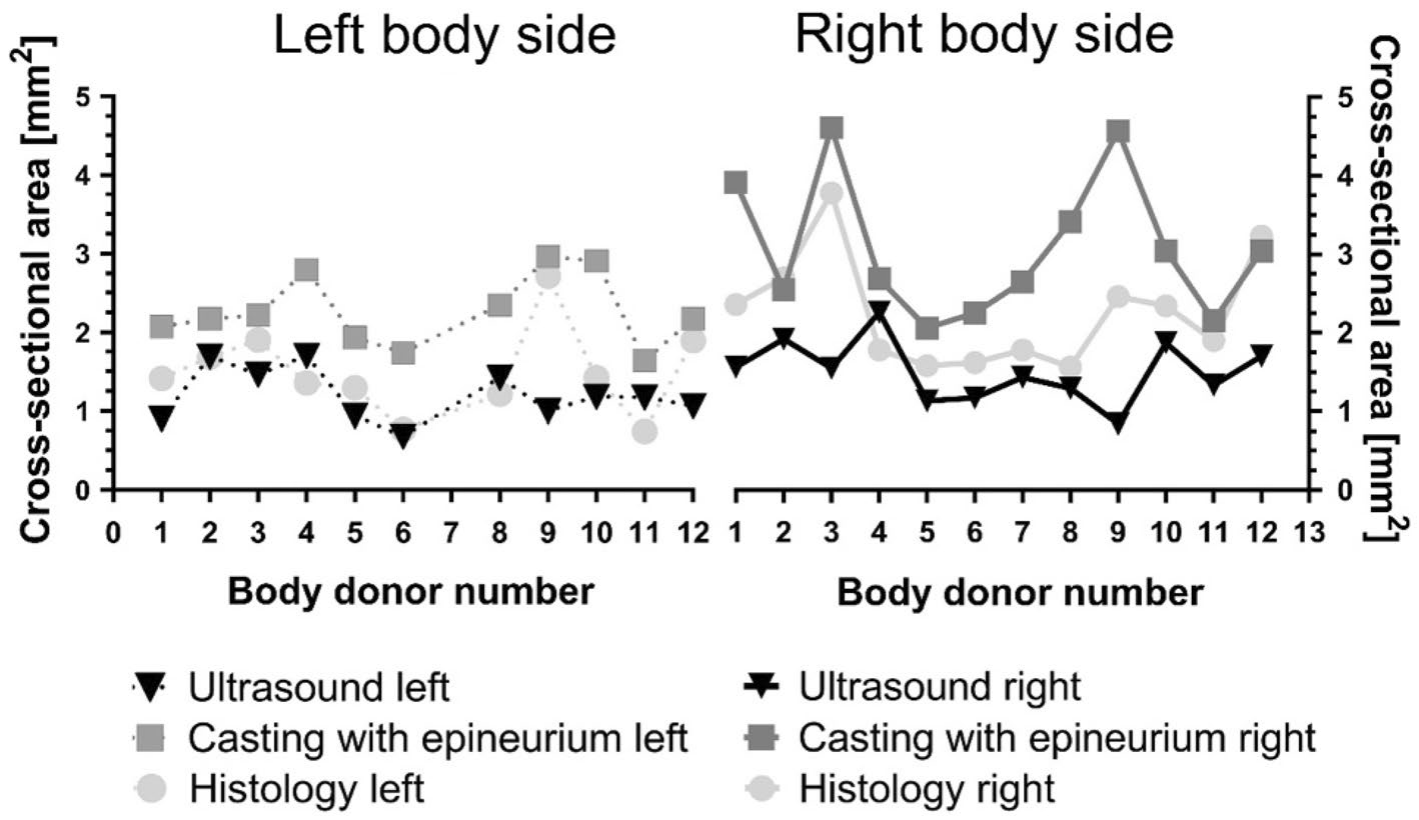
Determining the cervical vagus nerve cross-sectional area with ultrasound, cast, and histology. Interindividual differences donor- and side-depended were detected. Black—ultrasound darker grey—casting, lighter grey—histology. Dotted lines indicate the left and full lines the right body side.

Measurements including the epineurium gave consistently higher values than without epineurium. Ultrasound measurements including the epineurium showed an average CSA of 5.0 ± 1.1 mm^2^ (range 3.4–6.7 mm^2^; left; Fig. 4) and 5.6 ± 1.1 mm^2^ (range 4.0–7.0 mm^2^; right). In the histological measurements, the CSA with epineurium averaged 2.7 ± 1.0 mm^2^ (range 0.9–4.8 mm^2^; left) and 3.8 ± 1.2 mm^2^ (range 2.2–6.4 mm^2^; right), while the CSA of the axon tissue averaged 1.1 ± 0.3 mm^2^ (range 0.7–1.5 mm^2^; left) and 1.5 ± 0.4  mm^2^ (range 0.9–2.2 mm^2^; right), respectively.

CVN CSA yielded great interindividual differences comparing the measured values of different specimen. More information can be found in the Supplement figure and Table 1. Significant differences in the CSA were found, comparing ultrasound versus casting with epineurium (*p* ≤ 0.01) and ultrasound with epineurium versus casting with epineurium (*p* < 0.001). No significant differences were observed between ultrasound versus histology on the left (*p* = 0.414), but on the right side (*p* = 0.017) a significant difference was observed. Likewise casting with epineurium versus histology with epineurium yielded no significant difference on the left (*p* = 0.166) but on the right side (*p* = 0.020) a significant difference was observed. Strong correlations exist for casting with epineurium with histology with epineurium (left: r = 0.872, *p* < 0.001, right: r = 0.813, *p* = 0.001), while no significant correlation has been found for ultrasound with casting with epineurium (left: *p* = 0.163, right: *p* = 0.303), nor for ultrasound with epineurium versus casting with epineurium (left: *p* = 0.123, right: *p* = 0.084).

## Discussion

The aim of this study was to critically revisit high resolution-ultrasound measurements of the CVN as preoperative guidance for determining the nerve cross-sections in the context of VNS. While the clinical relevance for VNS grows^2,7,28^, further improvement in preoperative imaging is of relevance to choose best suited cuff electrodes, and potentially to reduce undesired side effects or lacking therapeutic efficiency due to poor cuff electrode fit^14^. Precise preoperative measurements of the CVN CSA could be a contributing factor in this process. Previous findings suggest sufficient validity and reliability for CSA measurements of peripheral nerves with ultrasound^19,26,29^. In contrast to these findings, this study is the first to compare clinical ultrasound of the CVN to its *in-situ* size and to histology-based measurements on the same tissues. It could be shown that CVN CSA and its greater and smaller diameters differed significantly between the methods (*p* ≤ 0.02).

Cartwright and colleagues compared ultrasound and manually obtained CSA measurements and found no difference between the methods^26^. In this given study, significant differences were found in CVN CSA measured in ultrasound versus casting with epineurium (*p* ≤ 0.01) and in ultrasound versus histology (*p* < 0.02). Every method used for CVN examination in this study has its own potential sources of error. More importantly, all of these methods differ in the ability to recognize of the epineurium. Ultrasound requires experienced skills identifying the nerve and capturing its CSA without distortion^19,26,30^. Following the convention by Schelle et al.^31^ in ultrasound, the CSA is measured sonographically within a hyperechoic rim, thus sparing the nerve’s epineurium in routine CSA measurements. This could lead to a potential underestimation of a cuff electrode size for VNS. Casting could manipulate the nerve due to its invasive manner^18^, while histology is biased by the fixation and embedding process, thereby causing shrinkage and distortion of the tissues^32^. In their histological analyses, Verlinden et al. reported differences measuring the CVN CSA with and without epineurium^33^.

Being a structure of rich and complex vascularity (Fig. 2), an undersized cuff electrode may interfere with sufficient blood supply, thereby building a potential risk for partial degeneration and nerval malfunctioning^11,34^. Furthermore, compressing the epineurium poses the nerve at risk of impaired function as an isolator, thereby resulting in undesired side effects of electrical stimulation^33^. Delayed vocal cord paralysis due to CVN coil loop compression and resultant ischemia has been reported^35^. Additionally, in a large retrospective review of manufacturer reported VNS complications, coil diameter was substantial in determining the incidence of vocal cord paralysis in adult patients^36^. Helmers and coworkers modelled mathematically the VNS, and found that the amount of epineurium is of critical importance prognosing the potential recruitment of fibers by electrical stimulation^37^. Comparing the CVN CSA in ultrasound (without epineurium) versus casting with epineurium this study reveals that in all cases ultrasound measurements were smaller compared to casting differentiating significantly in the size of the CSA (*p* < 0.01). Excluding the epineurium from CSA measurements in ultrasound might not lead to proper estimations of the CVN cross-section to implant a cuff electrode for VNS. In this study, various approaches have been applied to measure the amount of epineurium and to include the structure to ultrasound measurements. However, significant difference for ultrasound with epineurium versus casting with epineurium was found showing an invariably greater CSA and thereby a potential risk of overestimating the nerves’ cross-sections. Overestimating the cuff electrode size might likewise lead to insufficient electric transmission between the electrode and the nerve. This may affect VNS success rates, as a potential explanation for patients not receiving therapeutic benefit from VNS^3,12^. Preoperative ultrasound might not be a valid and reliable indicator to determine CVN CSA for choosing a matching cuff electrode size for VNS. Measurements just inside the hyperechoic rim might underestimate, while approaches including the nerve’s epineurium might overestimate the CSA of the CVN compared to casting.

**Figure 5:**
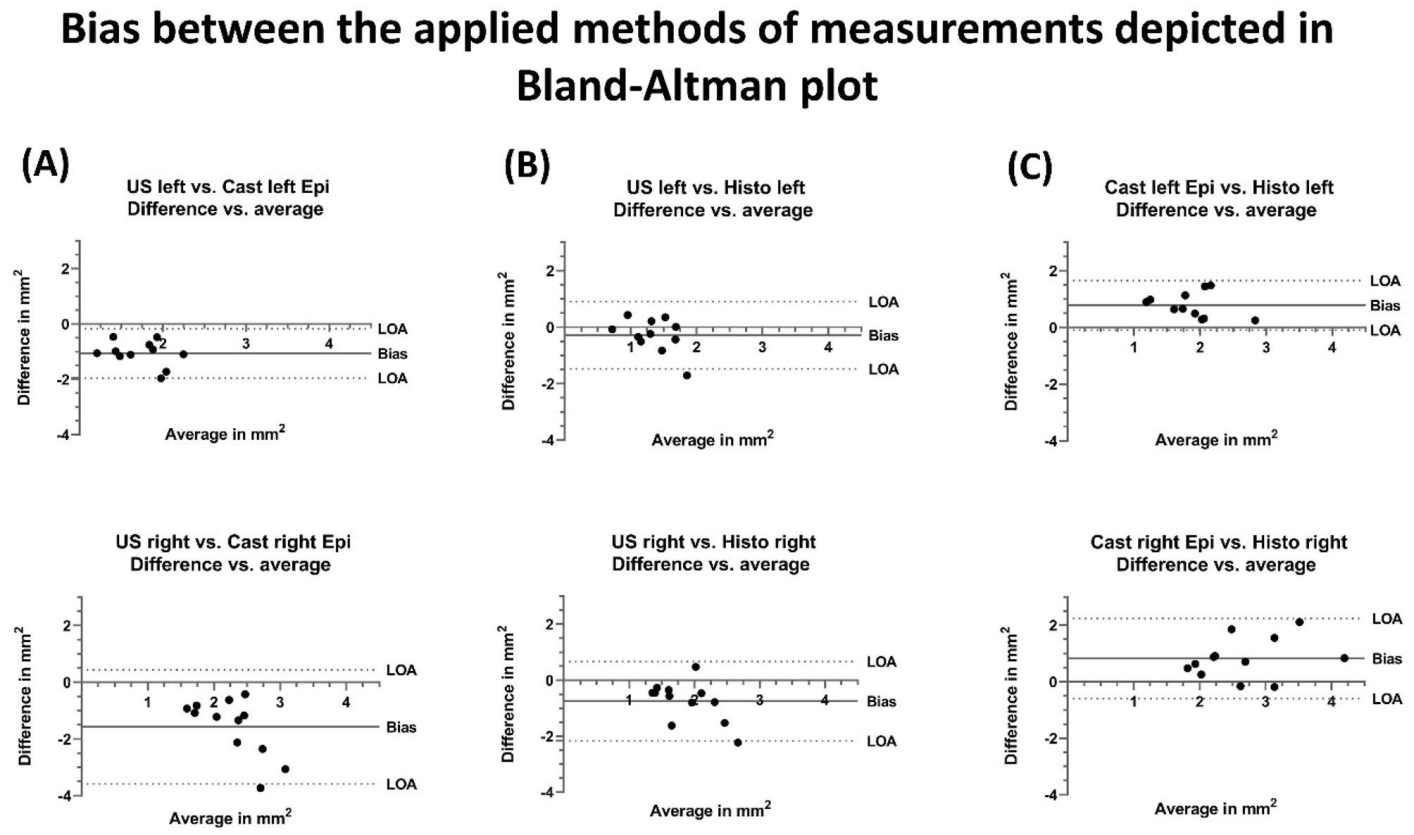
Bland–Altman plots showing the differences of measurement between the applied methods for cross-sectional area detection. Most values lied within the 95% confidence interval (LOA) yielding that differences are distributed within close borders. Ultrasound (US) and casting (Cast) with epineurium (Epi) showed negative bias (**A**), ultrasound versus histology (Histo) likewise a negative bias (**B**), and casting with epineurium versus histology a positive bias for both body sides (**C**). *LOA* Limits of agreement.

The casting measurements might more accurately reflect CVN CSA and diameters to individualize cuff electrode sizes to patient needs^12,13,16,38,39^. However, its invasive manner renders the clinical use of this method impossible. A correction factor to adjust for the systematic error between the methods could offer the possibility to estimate a matching size for a cuff electrode in VNS. The Bland and Altman analysis suggested a systematical error of underestimation between ultrasound and casting from 1 to 2 mm^2^ (Fig. 5). However, an application of a correction factor might only apply for the specific values measured using the individual ultrasound settings presented in this study.

Reviewing former studies on the CVN CSA, reference values for CSA measurements can be found (Table 2).

**Table 2:** Cross-sectional area (CSA) of the cervical vagus nerve (CVN) varies in different publications.

Author, year of publication	Method of measurement	Average CSA with standard deviation (mm^2^)	n	Average age (years)	Of Caucasian origin in %
Cartwright et al.^23^	Ultrasound	5.0 ± 2.0	60	45.9	92
Grimm et al.^24^	Ultrasound	2.2 ± 0.7	22	54.7	No information
Tawfik et al.^25^	Ultrasound	5.6 ± 1.4	20	46.1	100
Pelz et al.^19^	Ultrasound	2.4 ± 0.6	60	49.7	100
This study	Ultrasound	1.4 ± 0.6	12	88.7	100
	Ultrasound with epineurium	5.3 ± 1.1			

From Tawfik et al.^25^ only the group of healthy controls are given. The sample size is indicated by n.

Recent meta analyses declare cross-sections of the CVN between 2–3 mm^2^ as sonographic reference values in healthy adults^40,41^. However, great variation for the CVN CSA is shown in literature: Cartwright et al. measured an average CSA of 5.0 ± 2.0 mm^2^, Pelz et al. measured 2.4 ± 0.6 mm^2^, while 1.3 ± 0.6 mm^2^ were found in this study, respectively^19,23^. Firstly, all studies cited in Table 2 examined healthy patients. This study was the first to address high resolution ultrasound at native *post-mortem* tissues to measure the CSA of the CVN. *Post-mortem* autolysis may have had an impact on the CSA. Second, previous samples differentiated in several factors like age and ethnicity. Age has been described to influence the CSA of the CVN^19,42^ and on other peripheral nerves^26,43,44^. However, patients mean age in Pelz et al.^19,42^ and Cartwright et al.^19,33^ was similar, while mean CSA differentiated largely. Ethnicity, as far as known, was very similar in all cited studies. Cartwright et al. exclusively measured the CVN of the right side of the body^23^. Substantial difference in CVN CSA depending on the side has been described previously in histology and ultrasound^19,33^, while other studies found no significant difference in casting and ultrasound^18,25^. None of the here presented studies indicated to include the epineurium in their CSA measurements with ultrasound. In this study, CSA values differed greatly depending on the epineurium. Surprisingly, measurements of the CSA by Cartwright et al.^19,33^ and Tawfik et al.^25^ rather resemble the ultrasound measurements of this study including the epineurium than measurements excluding this structure.

The CVN CSA yielded large variation in literature. Several factors such as age and body side seemed to impact the CSA measurement. Nevertheless, the ultrasound setting (i.e., device, examiners experience, etc.) seems to add to the variation of measurements. It has been addressed that every ultrasound laboratory should define their own reference values^19,23^. Considering variable reference values depending on the setting of ultrasound examinations, a potential correction factor must be adapted to the individual setting of ultrasound measurements of every laboratory. Therefore, it seems that no individual value exists to correct for all possible differences in CSA introduced by different ultrasound settings. Future studies should focus on developing correction factors to their individual setting of ultrasound measurements with a greater sample size, and further refine ultrasound standards for the CVN to be able to provide accurate and reliable CSA values independent from the individual measuring laboratory.

This study only included a small sample with a limited age range between 71–101 years. In contrast to this, VNS is often used to treat infants^2^. Beside the specific age distribution, the small number of subjects and their homogenous origin need to be considered. The *post -mortem* interval between death and examination varied (12 h–4 days) due to administrative duties, leaving a possibility for autolyze effecting the native bodies. Another factor is that the results were based on individual data points, without replicating these data under different settings. Further studies should address these limitations in a larger sample size, for different age groups and more balanced group ratios (female vs. male) using ultrasound technology with higher frequencies (> 15 Hz). Future research should also consider the potential impact of aging on the CVN CSA since the CVN CSA was reported to decrease with increasing age^19^.

## Conclusions

In summary, the morphological data presented here questions the validity of high-resolution ultrasound to accurately reflect CVN size. An adjustment value to correct for the differences introduced by ultrasound, direct casting and histology may not only depend on the specific settings of ultrasound but also on tissue immanent characteristics such as the size of the epineurium and fascicle count.

## Methods

### Tissues

Twelve *post-mortem* bodies were examined in an unembalmed condition between April 2019 and March 2020. This cohort comprised nine females and three males (mean age 88.4 ± 8.5 years, range 71–101 years). Eleven cases were examined bilaterally and one unilaterally (previous surgery on the contralateral side). BMI averaged 27.7 ± 5.5 (range 19.5–33.8) kg/m^2^; the medical history and if available the cause of death were recorded. While alive, all body doners gave their written and informed consent to the *post-mortem* donation of their bodies for teaching and research purposes. Institutional approval for the use of the *post-mortem* tissues was obtained from the Institute of Anatomy, University of Leipzig. This institutional approval follows the Saxonian Death and Funeral Act of 1994^45^. All experiments have been conducted following the principles of the Declaration of Helsinki^46^.

**Figure 6:**
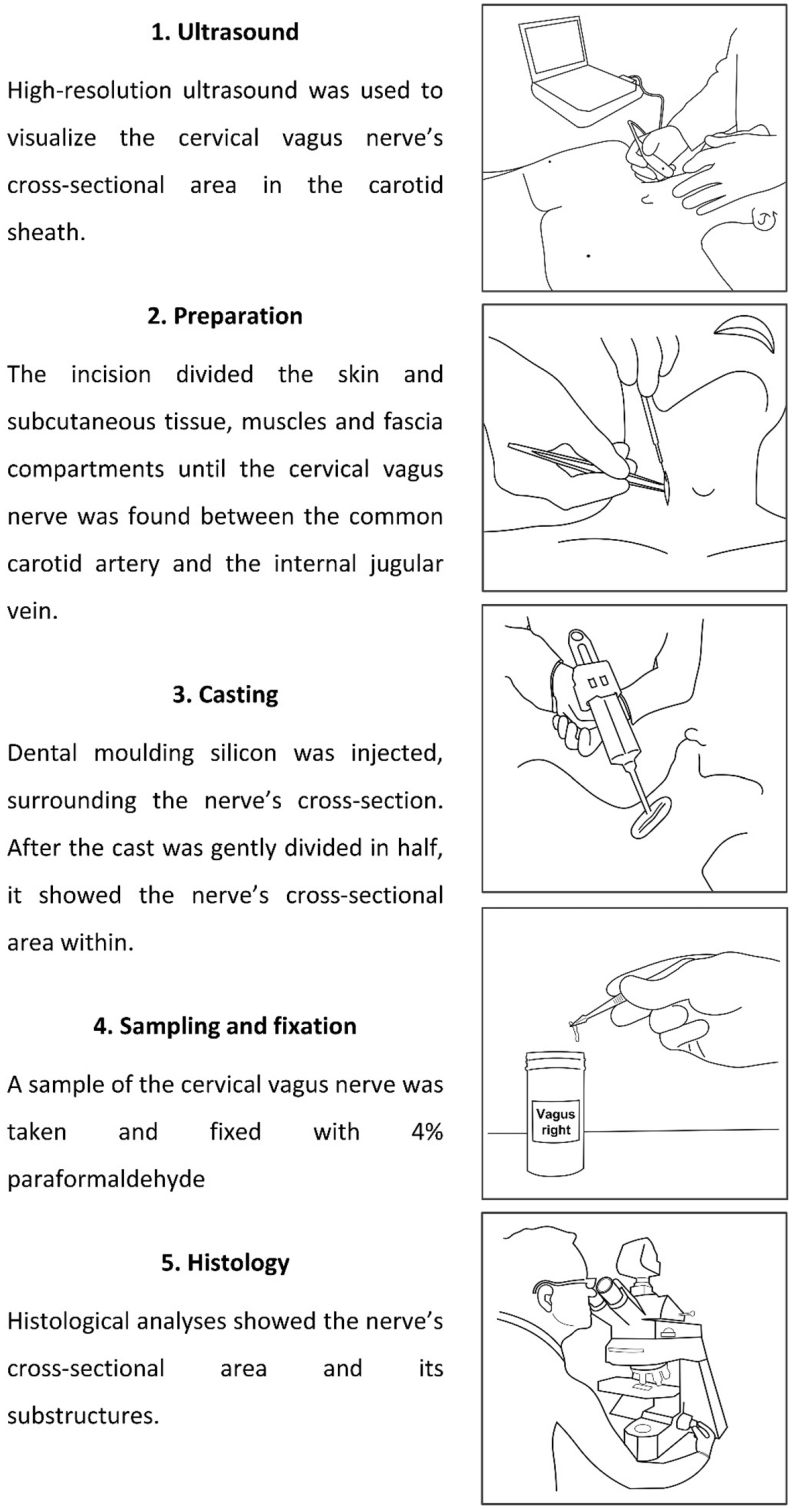
Five steps showing the workflow of analyzing the cervical vagus nerve’s cross-sectional area.

The cross-sectional area and the greater and lesser diameters of the CVN were obtained 10 mm caudal of the carotid bifurcation, using ultrasound, a method for manual casting and histological depiction. The examination was carried out in five steps (Fig. 6).

### Ultrasound of the CVN

**Figure 7:**
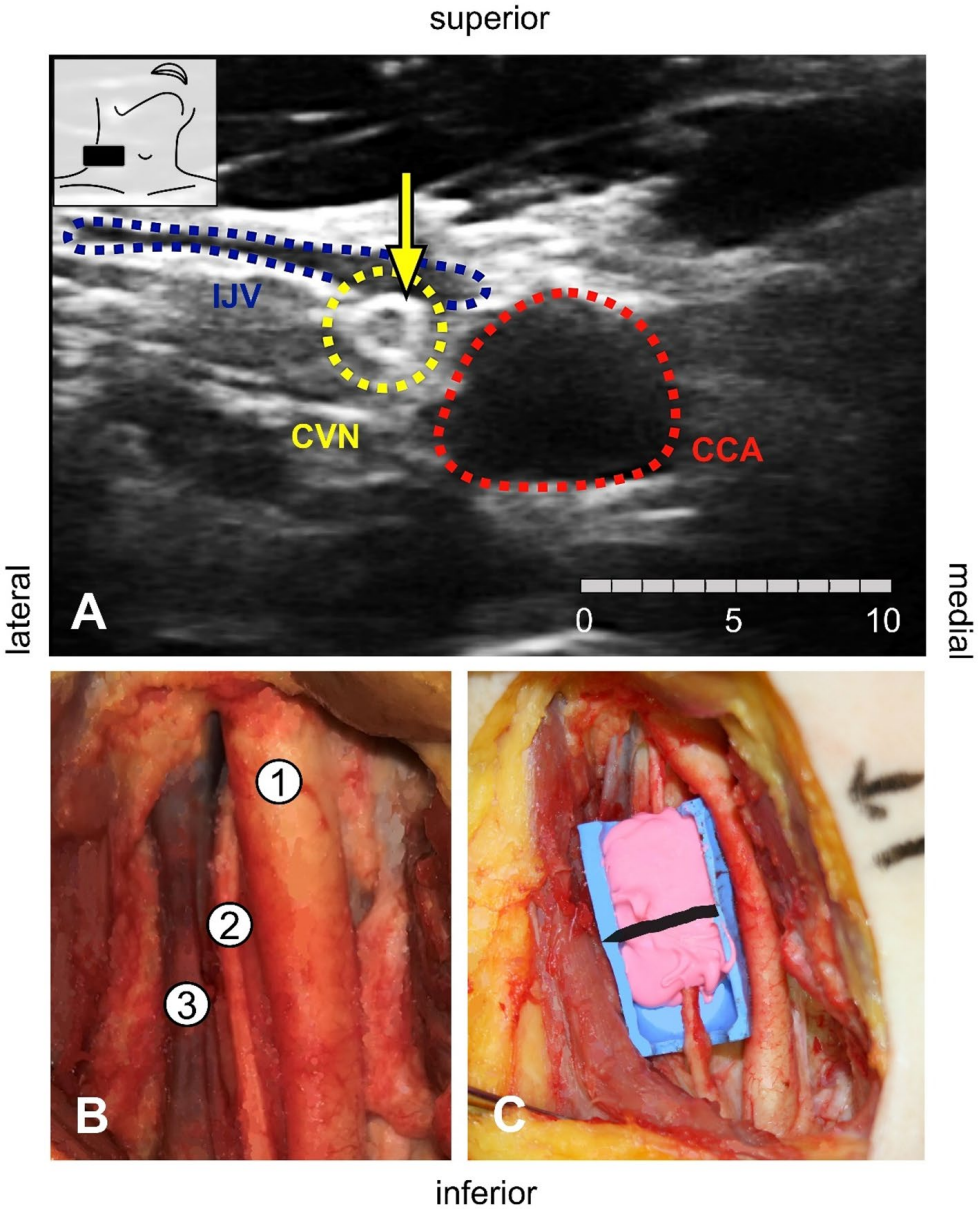
(**A**) The cervical vagus nerve (CVN), the internal jugular vein (IJV), and the common carotid artery (CCA) are shown using high-resolution ultrasound. The yellow arrow points out the nerve’s hyperechoic epineurium. The nerve’s cross-sectional area is measured within this structure (scale in mm). (**B**) Preparation of the common carotid artery (1), the cervical vagus nerve (2), and the internal jugular vein (3). Most often the nerve is found in between the great vessels. (**C**) Casting of the cervical vagus nerve with pink-colored dental molding silicon. The blue mold provides sufficient casting by separating the nerve from surrounding tissue and moisture. The black line marks the level of examination.

The CVN measurements were performed using an ultrasound system with a 15-MHz transducer (Esaote MyLab Five, Genova, Italy; probe no LA435). The bodies were placed in a supine position extending the neck slightly in a similar manner as described previously^19^. Ultrasound settings such as gain, depth, and focus were individualized for each specimen by the examiners. After identifying the CVN between the common carotid artery and the internal jugular vein, the transducer was placed perpendicular to the nerve’s course, ensuring the depiction of the smallest CSA (Fig. 7A). While positioning the transducer, minimal pressure was applied to prevent distortion of the CVN. Due to missing cardiovascular activity of the *post-mortem* bodies, the internal jugular vein was compressed with very low contact pressure. The CSA and the greater and lesser diameters of the CVN were obtained at the level of the thyroid gland (about 10 mm caudal of the carotid bifurcation). The exact level of ultrasound measurement was transferred to the skin with a waterproof pen. For offline measurements, the ImageJ software (version 1.52q, National Institute of Health, Bethesda, MA, USA; https://imagej.nih.gov/ij/download.html) was used. The examiner was blinded to all anthropometric information including age, gender, BMI, or body side during the measurements. For the CSA of the CVN only the nerve’s contour within a hyperechoic epineural rim was measured^31^. Another experimental approach was taken, measuring the nerves’ CSAs including a hyperechoic rim on the nerve’s outside. For this purpose, a freehand feature of ImageJ was used, allowing to depict the outer surrounding for the hypoechoic rim, combined with the invert color function to delineate the epineurium from surrounding tissues (Supplement Fig. S2). Three individual measurements were averaged from the same site and location for each of the values. An ICC of 0.978 indicates excellent measurement reliability for this approach.

### Preparation of the CVN and casting procedure

Preparation of the same CVNs assessed in ultrasound was performed in similar manner as for surgical exposure at the implantation of electrodes for VNS^10,11^. An incision was made parallel to the median sagittal plane about 20 mm lateral of thyroid cartilage in a supine position and extending the neck to the contralateral side. The skin and the subjacent platysma were transected. Adipose tissues were removed to enhance the exposure until the carotid sheath was reached. The incision was extended cranially to the posterior belly of the digastric muscle and caudally until contact to the omohyoid muscle^18^. The carotid sheath was found by shifting the sternocleidomastoid muscle laterally and following the sternohyoid muscle dorsally. After exposing the carotid sheath ventrally with scissors, the CVN was identified within (Fig. 7B). The CVN was carefully cleared of its surrounding connective tissue until the nerve’s surface had lost gloss. The site of the incision was held open by a spreader. The CVN was slightly lifted while a small, preformed molding template was installed beneath the nerve at the same level and height of the ultrasound examination (Fig. 7C). Polyvinyl siloxane (HS-A silicon; Henry Schein Inc., Melville, NY, USA) was injected into the mold surrounding the nerve’s cross-section^47^. After 15 min of curing, the CVN was cut at the mold’s rim and fixed in 4% paraformaldehyde for histological examination. To evaluate the influence of the fixation process, the CVN samples were casted again after fixation but prior to all other histological procedures. The casts of the CVN were divided in half, showing the nerve’s CSA, and scanned at 600 dpi. For digital measurements the ImageJ software was used. The average of three measurements was used for statistical analyses.

### Histological workflow

Tissue processing of the same specimens used for ultrasound and casting procedure was performed according to histological standard procedure^32^ for paraffin embedding with 4 µm sectioning on a rotary microtome (Leica RM2255, Nussloch, Germany). As derivate for xylol Histolab Clear (Sanova, Vienna, Austria) was used. Staining was done according to standard hematoxylin–eosin protocol^48^. Pictures were taken by transillumination microscope (Olympus BX43, 20 × Plan Fluorit Objective, Software: Olympus CellSense 3.1.1, Stacking Method: MIA, Olympus, Tokyo, Japan). Again, digital measurements were conducted with the ImageJ software, differentiating the measured area in the CSA with epineurium, axon tissue and stroma, and axon tissue. The average of three measurements was used for statistical analyses.

### Statistical analyses

For statistical analyses, Prism 9 (Graphpad software, La Jolla, CA, USA) and SPSS version 26.0 (IBM Corp, Armonk, NY, USA) were used. Following normality distribution assessment with the D’Agostino & Pearson test, comparisons were made using a one-way ANOVA for multiple comparisons without post-hoc correction due to the hypothesis-generating nature of this study. Comparisons of CVN cross-sections were made with epineurium (ultrasound, cast and histology assessment), without epineurium (ultrasound and histology assessment), and for the nerve only (histology assessment) to evaluate differences related to the various methods of cross-section data retrieval and side difference. Correlations with age, BMI and cross-section were analyzed side-dependently using Pearson correlations. For the latter, a two-way mixed model assessing for absolute agreement was chosen. *P* values of 0.05 or less were considered as statistically significant^49^.

## Supplementary Information

Supplementary Information.

## Data Availability

The datasets generated and analyzed during the current study are available in the supplements.
