# Supplementary material for: Comparing the accuracy of ultrasound-based measurements of the cervical vagus nerve
Source: Sci Rep. 2023 Jan 17;13:884. doi: 10.1038/s41598-023-27894-9 (PMC9845339; doi:10.1038/s41598-023-27894-9)
Supplement: Supplementary file 1 — Supplementary Information. [file 41598_2023_27894_MOESM1_ESM.pdf]

# Comparing the accuracy of ultrasound-based measurements of the cervical vagus nerve

Johann Dörschner[1], Johann Otto Pelz[2], Alexander Kerner[3], Jason Labuschagne[4], Niels Hammer[3,5,6,#,*], Sabine Löffler[1,#]

[1] Department of Anatomy, University of Leipzig, Germany

[2] Department of Neurology, University of Leipzig, Germany

[3] Division of Macroscopic and Clinical Anatomy, Gottfried Schatz Research Center, Medical University of Graz, Austria

[4] Department of Neuroscience, University of the Witwatersrand, South Africa

[5] Department of Orthopedic and Trauma Surgery, University of Leipzig, Germany

[6] Fraunhofer Institute for Machine Tools and Forming Technology (IWU), Medical Branch, Germany

[#]both authors contributed as equally

## *Corresponding author

Niels Hammer, M.D., Dr. habil., Division of Macroscopic and Clinical Anatomy, Gottfried Schatz Research Center, Medical University of Graz, 8010 Graz, Austria

Email: niels.hammer@medunigraz.at

# Supplements

**Supplementary Figure S1**
**Box plots comparing side difference in the cross-sectional area of the cervical vagus nerve**

Dotted boxes indicate the left side, striped boxes the right side. They show the 25%-75% percentile, whiskers the minima and maxima. The solid line marks the median. Black – ultrasound (US), darker grey – casting with epineurium (Cast), lighter grey – histology (Histo), Epi: Measurements contain the epineurium.

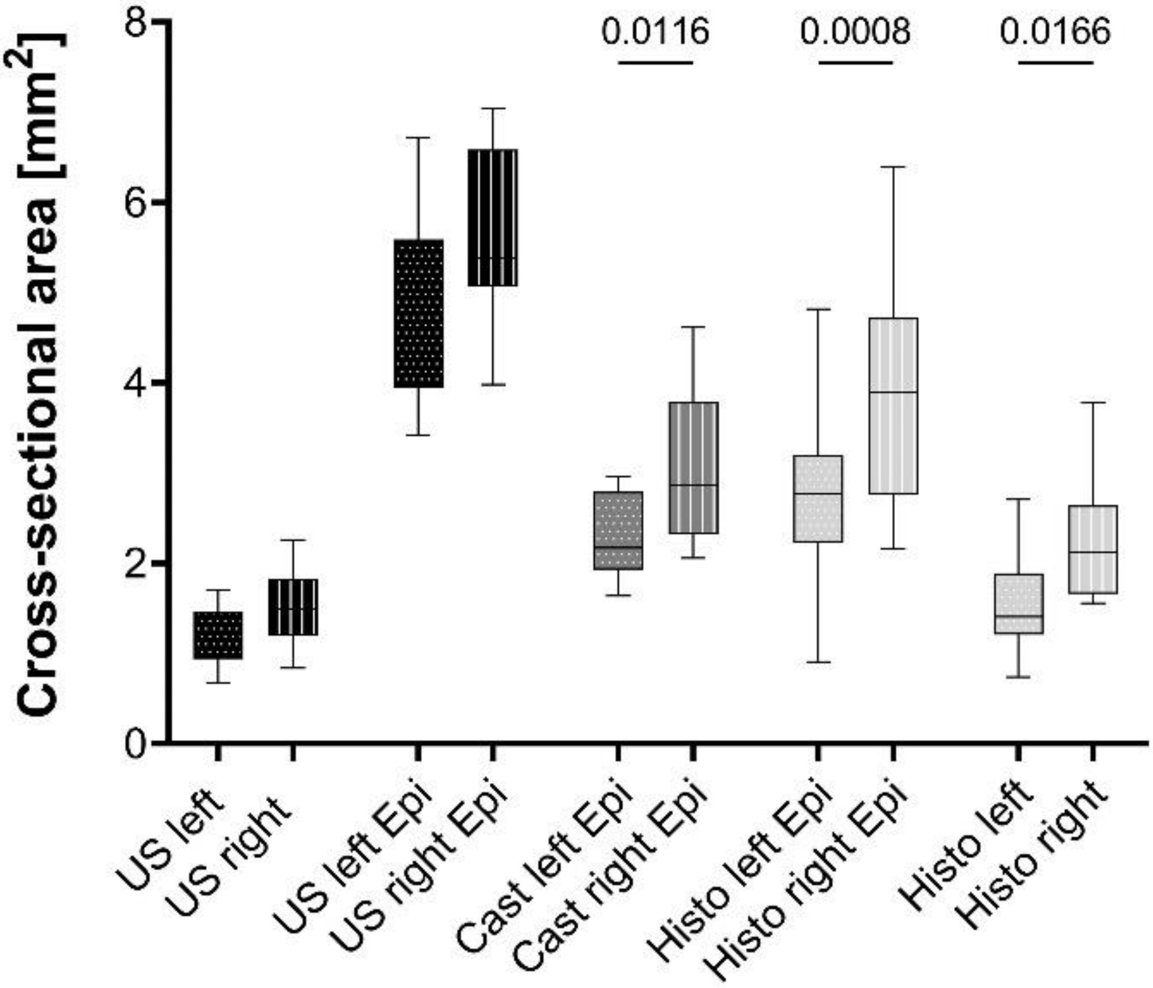

## Supplementary Figure S2

## Method for visualizing the epineurium of the cervical vagus nerve (CVN)

The cervical vagus nerve is highlighted in ImageJ using the free hand feature. Using the same data, the cross sections are determined without and with epineurium. ACC = common carotid artery, VJI = internal jugular vein, NX = vagus nerve. **(A)** Measurement without the epineurium, **(B)** measurement including the epineurium, **(C)** same dataset as shown in (B) using the inverted color function to delineate the epineurium from surrounding tissues.

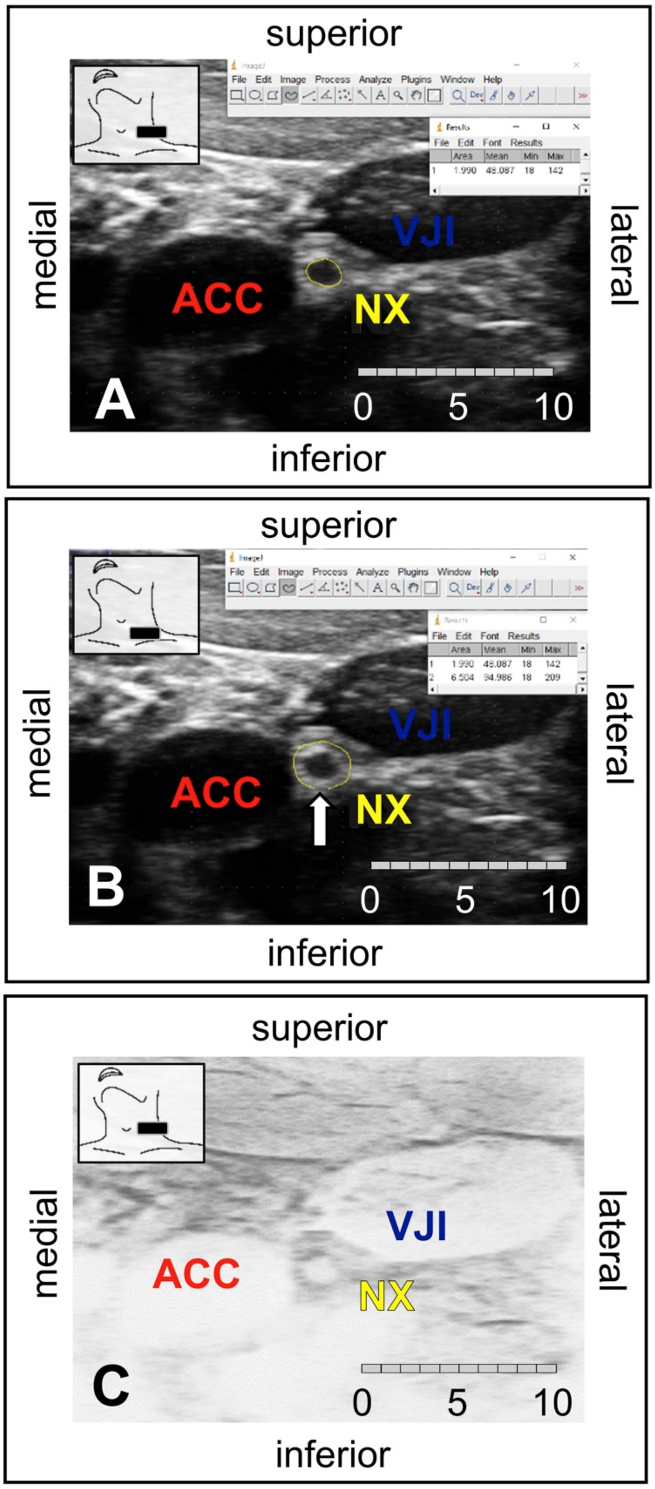

**Supplementary Table T1**

**The cross-sectional area of the cervical vagus nerve depends on the method of measurement and body side. Additional information concerning the body doners is given.**

| n=23 | Age [years] | Body mass index [kg/m²] | Side | Nerve fasicle count | Sex | Cross-sectional area ultrasound [mm²] | Cross-sectional area ultrasound with epineurium [mm²] | Cross-sectional area cast with epineurium [mm²] | Cross-sectional area histo [mm²] | Cross-sectional area histo with epineurium [mm²] | Cross-sectional area histo axon fibers [mm²] |
|---|---|---|---|---|---|---|---|---|---|---|---|
| **All** | 88.4 ± 8.5 | 27.7 ± 5.5 | 23 | 8.1 ± 3.7 | | 1.4 ± 0.4 | 5.3 ± 1.2 | 2.7 ± 0.8 | 1.9 ± 0.7 | 3.3 ± 1.2 | 1.3 ± 0.4 |
| **Maximum** | 101 | 19.5 | | 16 | | 2.3 | 7.0 | 4.6 | 2.7 | 6.4 | 2.2 |
| **Minimum** | 71 | 33.8 | | 3 | | 0.7 | 3.4 | 1.6 | 0.7 | 0.9 | 0.7 |
| **Left** | | | 11 | 6.3 ± 2.4 | | 1.2 ± 0.4 | 5.0 ± 1.1 | 2.3 ± 0.4 | 1.5 ± 0.5 | 2.7 ± 1.0 | 1.1 ± 0.3 |
| **Right** | | | 12 | 9.8 ± 3.9 | | 1.5 ± 0.4 | 5.6 ± 1.1 | 3.1 ± 0.9 | 2.3 ± 0.7 | 3.8 ± 1.2 | 1.5 ± 0.4 |
| **Females** | | | | | 9 | | | | | | |
| **Males** | | | | | 3 | | | | | | |

Values given in years, kg/m², and mm². Results with standard deviation. The sample size is indicated by n.
